# The Retina: A Window into the Brain

**DOI:** 10.3390/cells10123269

**Published:** 2021-11-23

**Authors:** Maurice Ptito, Maxime Bleau, Joseph Bouskila

**Affiliations:** 1 School of Optometry, University of Montreal, Montreal, QC H3T 1P1, Canada; maxime.bleau.1@umontreal.ca (M.B.); joseph.bouskila@umontreal.ca (J.B.); 2 Department of Neuroscience, Copenhagen University, 2200 Copenhagen, Denmark; 3 Department of Neurology and Neurosurgery, Montreal Neurological Institute, McGill University, Montreal, QC H3A 2B4, Canada

In the course of evolution, animals have obtained the capacity to perceive and encode their environment via the development of sensory systems such as touch, olfaction, audition, and vision. In many vertebrate species, vision is the most predominant sense for accumulating and perceiving environmental information. Vision plays a pivotal role in many interactions with the environment and with other living organisms. The eyes have adapted to complex environments, enabling animals to effectively navigate, procreate, forage for food, hunt for prey, or shelter from predators (reviewed in [1,2]).

The mammalian eye is composed of three concentric and distinct layers of tissue: the sclera, the uvea, and the retina. It is a laminar tissue containing neurons and glial cells that plays the crucial role of phototransduction, the process of converting light energy into encoded neural signals delivered to the brain [3,4]. The retina has a complex laminar organization and a cellular composition that are similar in all vertebrates. However, the layout of the retina varies between species to meet their specific needs, behaviors, and habitat (Figure 1). Humans and other primates need to identify food and predators against cluttered environments, and consequently evolved a zone of acute vision located at the center of the retina, namely, the macula. This area (5.5 mm in diameter) is specialized for color and detailed vision enabled by its higher density of cones and ganglion cells [5]. At the macular center is located the fovea, a zone with a 700 µm deep focus approximately 1.5 mm in diameter [2], composed exclusively of cones, thus providing the highest resolution for daytime vision. Nasally to the macula is located the optic disc; in this zone, all ganglion cell axons converge to exit the eye *en route* to the visual brain via the retinofugal pathways [6]. Due to this retinal configuration with a central zone of higher visual acuity extending over a limited field of view, humans and other higher primates have developed highly motile eyes [7,8,9]. This retinal organization and associated visual behavior had significant consequences for the evolutionary development of the occipital, frontal and prefrontal cortical areas in the simian lineage, as vision became the most important sensory system.

The human retina is a complex mosaic (Figure 2B,C) comprised of five classes of neurons specialised in processing the visual information received by the eye. These neuron types are the photoreceptors, bipolar cells, ganglion cells, horizontal cells and amacrine cells [4]. These neurons have a characteristic distribution in the ten distinct layers of the retina, and have interconnections via two different pathways, namely, the vertical and horizontal pathways. Their extensive interconnections enable the processing of the visual image projected on the retina and its transmission to the brain through the optic nerve. The vertical pathway begins with the transduction of light signals by the photoreceptors and ends with transmission to the brain via the axons of retinal ganglion cells (RGCs). The horizontal pathway is comprised of horizontal cells and amacrine cells that connect laterally to provide feedback and feedforward signals between photoreceptors and bipolar cells (for horizontal cells) and between bipolar cells and RGCs (for the amacrine cells) [10]. The horizontal pathway mediates photoreceptor convergence, motion processing and contextual modulation [11]. Therefore, these two pathways embody the basic characteristics of retinal architecture and, hence, visual processing through their connections within the retinal layers.

The ten layers of the retina (Figure 2B,C), proceeding from the innermost to the outermost, are traditionally named as follows: the inner limiting membrane, nerve fibre layer (NFL), ganglion cell layer (GCL), inner plexiform layer (IPL), inner nuclear layer (INL), outer plexiform layer (OPL), outer nuclear layer (ONL), external limiting membrane, photoreceptors layer (PL), and the retinal pigment epithelium (RPE). The RPE is composed of epithelial cells with a rich content of melanosomes and melanin granules. RPE cells support the metabolic activity of the retina and supply the photoreceptors with nutrients and oxygen [12,13,14]. The photoreceptor layer is composed of the photosensitive outer segments and inner segments of the photoreceptors, the rods and cones, which are specialized for capturing and transducing light energy into electrochemical signals. The next layer, the outer limiting membrane, is formed by the extensions of large glial cells, known as the retinal Müller cells. The fourth layer is the outer nuclear layer, which contains the photoreceptors somata and nuclei. The photoreceptors then make synaptic contact with the bipolar and horizontal cells in the fifth layer, forming the outer plexiform layer. The sixth layer, the inner nuclear layer, is composed of cell bodies of horizontal, amacrine and bipolar cells. The latter cell type makes synapses with amacrine and ganglion cells in the inner plexiform layer. Ganglion cells bodies then form the ganglion cell layer, where their long axons run horizontally along the nerve fiber layer towards the optic disk. The tenth and final layer, the inner limiting membrane, is formed by extensions of Müller cells.

The vertical pathway (Figure 3) is defined by the capture and transduction of photons by the photoreceptors and the transmission of the resulting electrical signal to RGCs via their connections with bipolar cells. The main neurochemical involved in this pathway is glutamate, an excitatory neurotransmitter. There are two types of photoreceptors, the rods and cones, which both possess outer segments that are composed of stacked disks of infolded membranes containing the visual photopigments (opsin or rhodopsin coupled to a chromophore) [17]. Rods and cones differ not only in shape but also with respect to the composition of their outer segment disks, light and spectral sensitivity, and convergence towards RGCs. Rods are thinner (averaging 2 µm) and longer (averaging 50 µm) than cones. The functional particularity of rod outer segments derives from their abundant photopigment disks [18]. This property imparts greater light absorption capacity and, thus, higher light sensitivity compared to cones [13]. Moreover, through the retina, rods have a greater degree of convergence towards RGCs through their connections with bipolar cells, which serves to provide greater signal amplification, but with lower visual acuity. As such, rods are responsible for scotopic vision, but are saturated during the day or in other situations of high luminosity [19].

Cones, on the other hand, are generally thicker (3 to 5 µm) and shorter (40 µm) than rods and have a lower degree of convergence; this ratio even reaches 1 RGC for 1 cone in the fovea. The less numerous photopigment disks of the cones float freely in their outer segments [20,21]. Being less sensitive to low levels of light and only activated in situations of high luminosity, cones mediate photopic vision, operating when rods are saturated. However, both cones and rods are active in intermediate lighting conditions, such as daybreak and twilight [22]. Cones are also responsible for high-acuity vision, based on their 1-to-1 ratio of convergence in the central retina. Furthermore, three types of cones (S, L and M cones) are present in the human and nonhuman primate retina and contain the three types of opsins with different spectral sensitivity [3,23,24].

Populations of cones and rods have different distributions throughout the retina (Figure 2D). Typically, cones are outnumbered by rods by a ratio of 20- or 30-to-1 [3]. The human retina contains approximately 90 to 120 millions rods and 5 to 7 millions cones [16,25]. Throughout the peripheral retina, the density of rods greatly exceeds that of cones. However, this ratio is shifted in the fovea, where the density of cones increases almost 200-fold [25]. In that region, the individual cones are thinner (Figure 3B), allowing for the highest photoreceptor density recorded in the retina [13,25]. As a result, the central retina mediates photopic vision with a high degree of resolution (or visual acuity) and color perception, while the periphery is responsible for scotopic vision and motion sensitivity, albeit with lower visual acuity. Both types of photoreceptors send signals to the parallel pathways of bipolar cells (reviewed in [3]) via the release of glutamate. 

Bipolar cells (Figure 3C) are the interneurons linking photoreceptors to RGCs and amacrine cells. Morphologically, bipolar cells are recognized by their two protrusions, one extending in the outer retina and making synaptic contact with photoreceptors and horizontal cells, and the other protrusion extending in the inner retina as the axon that relays signals from the photoreceptors to RGCs and amacrine cells. There are diverse morphological types of bipolar cells, of which the axons terminate different levels, or strata, of the inner plexiform layer. Bipolar cells thus contact different types and sets of RGCs and amacrine cells (reviewed in [26]). There are 12 types of cone bipolar cell types, and only one type of rod bipolar cell that relay the signals from rods at low light intensities [27,28,29,30]. Bipolar cells are divided into ON and OFF types, thus subserving the first step in encoding visual information according to the intensity of light received by photoreceptors [26,31,32].

In the second synaptic layer of the retina, the inner plexiform layer, bipolar cells make synaptic contact with the dendritic arborizations of the third-order neurons, namely, the RGCs (Figure 3D). These cells have relatively large somata located in the ganglion cells layer that give rise to long axons extending horizontally in the nerve fiber layer and exiting the eye through the optic nerve. As such, the RGCs are the direct portal from the retina to the brain. Numbering around 0.7 to 1.5 million in the human retina [33,34,35], RGCs are separated from each other by glial processes of Müller cells. They are arranged in a single cell layer, except at the macula, where the ganglion cell layer is about 8 to 10 cells thick and contains 50% of all RGCs due to the far lesser photoreceptor convergence [2,36]. RGCs were traditionally classified as ON- and OFF-center RGCs, responding to increases or decreases in light intensity presented at the center of their receptive field [37]. However, there are many subtypes of RGCs differentiated by morphological criteria (e.g., dendritic arborisation), functional criteria (response to different stimuli) and molecular criteria [38,39,40]. There are also five types of intrinsically photosensitive RGCs (ipRGCs), which were discovered only recently [41]. These light-sensitive cells express melanopsin, which, upon stimulation by light, activates a signaling cascade that hyperpolarizes the neuron. ipRGCs mostly project to the suprachiasmatic nucleus and thus contribute to the synchronization of the circadian oscillator [40,42,43]. There are also three types of alpha RGCs [44], three types of Local Edge Detectors RGCs [45] and three types of J-RGCs expressing junctional adhesion molecule B [46]. Because of this high diversity of ganglion cell types, each sensitive to different visual features, the retina is not a simple relay structure but the first center of complex visual processing, sending preprocessed images of the external world to the brain.

The horizontal pathway (Figure 4) is comprised of two types of interneurons, horizontal and amacrine cells, which together subserve the connectivity and complementarity of subparts of the vertical circuitry at two different levels of the retina (OPL and IPL). This cellular architecture establishes the interconnected mosaic that defines the vast array of retinal image-processing functions. Indeed, horizontal and amacrine cells are essential for the creation of the center-surround properties of RGC receptive fields, along with many other visual functions observed in the retina, such as surround inhibition of photoreceptor cells [3,10,33]. The main chemical involved in this pathway is gamma aminobutyric acid (GABA), an inhibitory neurotransmitter. Horizontal cell types (Figure 4B) are distinguishable by morphological criteria and molecular markers [47]. The main classification is into A-type, or axon-bearing horizontal cells, and B-type, or axon-less horizontal cells [47]. Both subtypes are GABAergic interneurons, providing inhibitory feedback to cones or rods.

Amacrine cells mainly extend laterally (but some vertically) in the inner plexiform layer and receive inputs from bipolar cells (Figure 4C). Amacrine cells are astonishingly specialized interneurons that send feedforward signals to RGCs, feedback signals to bipolar cells and even inhibitory signals to other nearby amacrine cells (reviewed in [10,33]). Indeed, amacrine cells are inhibitory interneurons that mediate the spatial and temporal characteristics of RGCs’ receptive fields and light responses [48,49], refine the center-surround receptive fields of bipolar cells [50], sharpen the bipolar cell responses timing [51] and can regulate the gain of feedforward signals [52]. Amacrine cells, similar to horizontal cells, express the atypical endocannabinoid receptor TRPV1 [53].

The numerous subtypes of amacrine cells are all inhibitory (GABAergic or glycinergic, along with a secondary neurotransmitter such as acetylcholine) and are mostly categorized functionally into narrow-, medium- and wide-field amacrine cells, according to the size of their dendritic arborization [10,33]. Wide-field amacrine cells are mostly GABAergic interneurons with arborizations that extend in diameter from 100 µm (average 350 µm) to the millimeter scale [54], whereby they mediate long-range interactions as well as inhibitory surrounds in RGCs [3,55,56]. Starburst amacrine cells are a subtype of GABAergic/cholinergic wide-field amacrine cells that impart direction of movement selectivity in some RGCs [33,57]. Medium-field amacrine cells, of which there are at least eight types, including spiny AC, secretoneurin AC are GABAergic/glycinergic, have stratified dendritic arborizations extending between 100 and 500 μm. These cells gather and distribute signals across multiple levels of the IPL [33]. Finally, narrow-field amacrine cells, of which there are at least nine types, have dendritic arborization less than 100 μm wide and are commonly glycinergic interneurons [58,59].

The intricate organization of the retina makes it the perfect window into the brain. Unsurprisingly, vision is the dominant sense in many species, such that 30 to 55% of the brain is devoted to vision, depending on the species [62]. As a result, any loss of retinal function will have dire consequences, extending from various perceptual impairments to blindness (see Table 1). Indeed, many visual problems diagnosed in patients arise due to a disorder of the retinal mosaic. Most human retinal diseases mainly affect one of two sites in the human retina: the photoreceptor layer and the RPE or RGC and nerve fibers layers. The RPE and photoreceptors layers are the main locus of serious pathologies such as Age-Related Macular Degeneration (AMD) and autosomal disorders such as Retinitis Pigmentosa (RP), Juvenile Macular Degeneration (Stargardt’s disease and Best’s disease), achromatopsia and retinal detachment. AMD is the most prevalent cause of visual loss in older adults around the globe. It is characterized by the deposition of lipids and proteins (Drusen) in RPE cells, along with the degeneration of photoreceptors, especially in the macula region (reviewed in [63]). The onset of AMD is marked by progressive loss of acuity in the central vision (scotoma), which impedes important visual functions such as reading and face recognition. RP is an autosomal-dominant retinal dystrophy marked by the progressive loss of RPE cells and apoptosis of photoreceptor cells, causing progressive vision loss, extending from the peripheral to central vision (i.e., ring scotoma or tunnel vision). Peripheral vision loss often leads to night blindness and challenges during locomotion, such as collisions with unseen obstacles. Stargardt’s disease is an autosomal recessive disorder affecting photoreceptor cells and causing vision loss at an early age, whereas Best’s disease (or vitelliform macular dystrophy) is an autosomal dominant disorder the leads to protein/lipid deposits between the RPE and photoreceptor layers [64]. Furthermore, achromatopsia, or color blindness, is an X-linked form of congenital cone dystrophy, leading to improper light transduction and reduced perception [65]. The junction between the RPE and photoreceptor cell layers is also a common site of retinal detachment. RGCs and their axons (GCL and NFL) are also commonly affected in glaucoma and other retinopathies (i.e., diabetic, hypertensive and retinopathy of prematurity) associated with micro-haemorrhages in the NFL. Glaucoma, which usually results from increased intraocular pressure, is marked by the degeneration of RGCs, leading to significant losses in visual field and contrast sensitivity [66].

Vision is undoubtedly the most important sense for humans, and visual impairments have dire consequences for quality of life and productive activities [67,68,69,70]. The retina contains a surprising complexity in its cellular architecture, and literally presents a window to the brain; no other part of the central nervous system is amenable to direct observation. Because of the importance of human vision, researchers are making a broad effort to understand better the complex details of retinal function, even to the extent of developing highly invasive cybernetic inputs replacing the retina, or advanced methods for behavioral sensory substitution [71,72,73].

Therefore, this Special Issue of *Cells* is timely and brings a collection of groundbreaking novel research on the cellular and molecular aspects of healthy and diseased retinas. We present here 20 original articles and six reviews from the international research community, all with the goal of advancing knowledge towards the prevention and cure of visual pathologies, mainly AMD, RP and diabetic retinopathy. Papers in this Special Issue can be regrouped into five major themes. 

The first group of articles focuses on the neurovisual development of the retina in rodents and birds. Laroche et al. bring interesting results on the role of L-lactate and its receptor GPR81 on the growth of RGC axons during development in mice. They show that treatment of axon growth cones with L-Lactate or GPR81 agonist increased the cones size and number of filopodia. The following two papers study the role of cell death (autophagy and apoptosis) in the postnatal development of the retina. Pesce et al. show increased autophagy levels in the early retinal hypoxic phase and normalized levels in the mature, fully vascularized retina, and Álvarez-Hernán et al. propose that programmed cell death markers during early retinal development in hatched altricial birds is a potential model to study post-natal retinal development.

The second group of articles deals with diabetic retinopathy, its ischemic and hemorrhagic mechanisms, related markers and possible therapies in many species, including man. Shaw et al. investigate the use of quantitative Optical Coherence Tomography Angiography (OCTA) to track systemic vascular functioning and microvascular complications in black patients with diabetes mellitus. Adeghate et al. examine the effects of early onset diabetes on the retinal ultrastructure and cellular bioenergetics in rats. They found an increase in incretins and antioxidant levels as well as increased oxidative phosphorylation, which are events that may transiently preserve visual function. Kim et al. investigate the inhibition of Drp1, its diabetes-induced overexpression being linked to mitochondrial dysfunction and apoptosis of retinal endothelial cells, with the goal to protect the retina against vascular lesions in diabetic retinopathy. Musayeva et al. studied the neuroprotective effects of betulinic acid against ischemia and reperfusion injuries, often related to pathologies such as diabetic retinopathy and glaucoma, in the mouse retina. They showed that the injection of betulinic acid improved endothelial functioning and caused a reduction in reactive oxygen species (ROS) levels following ischemia. Middel et al. propose a review about studies on the zebrafish neurovascular unit in the context of diabetic retinopathy and discuss the advantages of the zebrafish model in studying this specific pathology. As for ischemic injuries, Agrawal et al. tested iSMC as a potential therapy for reducing inflammation and retinal degeneration.

The third group of articles focuses on changes and markers associated with retinal neurodegenerative diseases and on potential therapies. Hamid et al. explore the effects of anti-VEGF drugs on different AMD genetic markers in retinal epithelial cells. Holan et al. review the literature on mesenchymal stem cell (MSC) therapy for many retinal degenerative diseases, such as AMD, RP, glaucoma and diabetic retinopathy. They provide insights on MSCs’ therapeutic properties, such as the production of growth/neurotrophic factors. Othman et al. propose an interesting review on the kallikrein-kinin system (KKS), its important role in neurodegenerative diseases such as AMD and diabetic retinopathy, and how it provides promising therapeutic targets against these diseases. The last two original articles in this section explore changes and markers in neurodegeneration induced by light in rodents. Riccitelli et al. investigate the time course of retinal changes (neurodegeneration and recovery) following light exposure in rats and suggest the development of diagnostic tools to monitor the progression of pathologies and efficacy of treatments. Park et al. investigate the expression of stress markers in retinal degeneration induced by blue LED stimuli. They show that these markers may serve an important role in the activation of glial cells during degeneration of photoreceptors. Rajendran et al. underline the therapeutic potential of RPE grafts during long-term observations in a rat model of RPE. Candadai et al. conclude on the neuroprotective effects of Fingolimod in an in vitro model of optic neuritis. Girol et al. demonstrate that in a case of endotoxin-induced uveitis, the inflammation can be treated with piperlongumine and/or Annexin A1 mimetic peptide (Ac2-26). 

The fourth group of articles explores the role of glial cells in retinal function. Lee et al. investigate the response of Müller cells and microglia in retinal detachment. They found that glial activation markers were differently expressed in intact and detached regions. Choi, Guo and Cordeiro propose a review exploring the role and morphology of retinal microglia as well as their activation by stress stimuli in multiple sclerosis and other neurodegenerative diseases such as Alzheimer’s disease, Parkinson’s disease, glaucoma and RP. Finally, Yoo et al. present an interesting review on the role of retinal astrocytes and Müller cells and the potential molecular pathways that can induce these cells to become growth-supportive and thus promote the survival and regeneration of RGCs in neurodegenerative diseases.

The fifth and final group of articles brings novel data on the molecular and physiological aspects of visual function. Solanki et al. investigate the role of the motor protein MYO1C, an unconventional myosin, and how its loss causes the mis-localization of rhodopsin in photoreceptors, leading to impaired visual function in mice. Dao et al. bring novel data on high-fat diets associated with the development and progression of many retinal diseases, such as AMD and diabetic retinopathy. They show that such diets can alter the retinal transcriptome independently of gut microbiota. Fusz et al. investigate the variation in gap junction connections across the mammalian (mouse, rat and cat) retina via the topographical distribution of connexin-36 (associated with electrical synapses) in ON and OFF amacrine cells. Bouskila et al. present a review on the expression, localization and function of endocannabinoids in the horizontal and vertical pathways of the monkey’s retina and discuss their potential as therapeutic targets for visual pathologies such as AMD, glaucoma and diabetic retinopathy. Piarulli et al. report a case of a patient with Charles Bonnet syndrome, a health condition characterized by vivid visual hallucinations in individuals with retinal vision loss. Dumitrascu et al. showed that retinal curcumin-fluorescence imaging is a good predictor of verbal memory loss linked to abnormal retinal vasculature and amyloid count. Finally, long exposure to short wavelength LED light causes retinal damage that can be reduced by the use of short wavelength selective filters apposed on the LED screens, as demonstrated by Sanchez-Ramos et al.

We sincerely hope that the papers presented in this Special Issue of *Cells* will contribute to the understanding of retinal diseases and their underlying mechanisms and will lead to the development of new pharmaceutical tools to treat visual disorders.

## Figures and Tables

**Figure 1:**
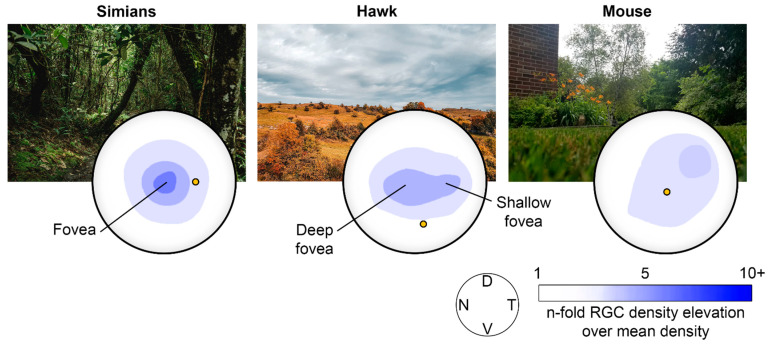
Snapshots of the habitat of different species along with their representative RGC density distribution across their retinal surfaces. (**Left**) Habitat of simians (jungle) and the RGC density distribution in the human retina. (**Center**) example of a typical habitat of the hawk (sky) and its retina RGC density distribution. (**Right**) example of a typical habitat of the mouse (ground) and its retina RGC density distribution. D, dorsal; N, nasal; T, temporal; V, ventral. Redrawn from [1].

**Figure 2:**
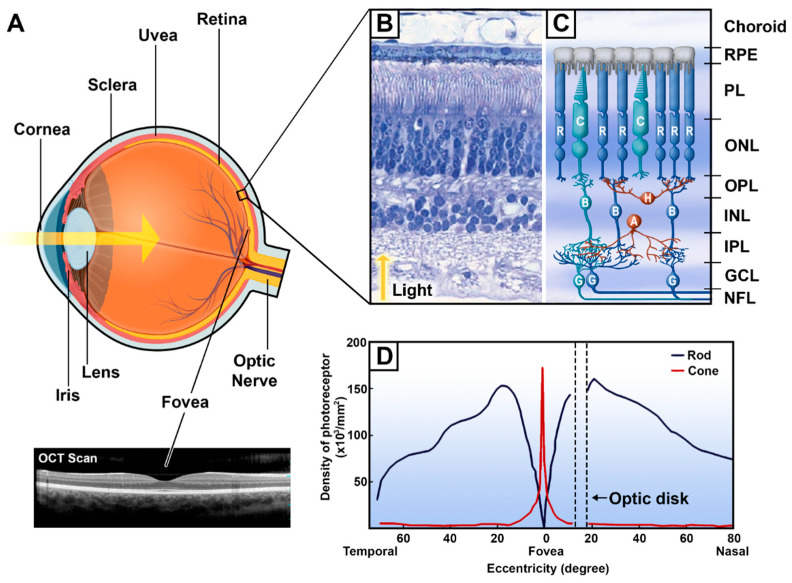
The eye and the retinal mosaic. (**A**) A sagittal section of the eye (created with Biorender.com) and an Optical Coherence Tomography (OCT) scan of the central retina. The yellow arrow represents the path of the light rays entering the eye. (**B**) An H&E-stained transverse section of a human retina. Adapted from [15]. (**C**) Schematic organization of retinal cells from the retinal pigment epithelium to the nerve fiber layer. (**D**) Density of cones and rods throughout the retinal surface. Adapted from [16]. OCT, optical coherence tomography; R, rod; C, cone; H, horizontal cells; B, bipolar cells; A, amacrine cells; G, retinal ganglion cells; RPE, retinal pigment epithelium; PL, photoreceptor layer; ONL, outer nuclear layer; OPL, outer plexiform layer; INL, inner nuclear layer; IPL, inner plexiform layer; GCL, ganglion cell layer; NFL, nerve fiber layer.

**Figure 3:**
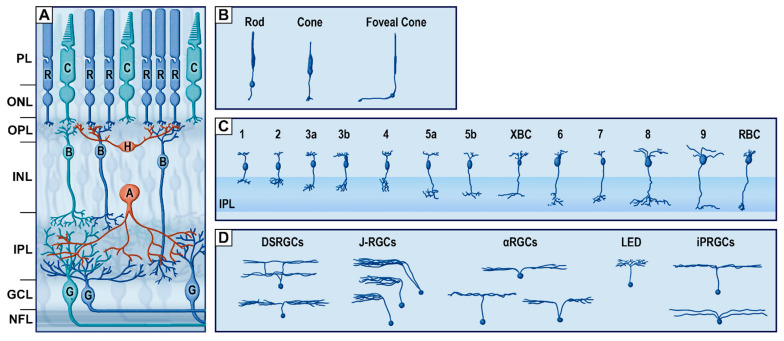
The vertical pathway of the retina. (**A**) Schematic organization of retinal cells. (**B**) Subtypes of photoreceptors (redrawn from von Greef, 1900). (**C**) Subtypes of bipolar cells. Redrawn from [26]. (**D**) Subtypes of retinal ganglion cells (RGCs) (redrawn from [38]). PL, photoreceptor layer; ONL, outer nuclear layer; OPL, outer plexiform layer; INL, inner nuclear layer; IPL, inner plexiform layer; GCL, ganglion cell layer; NFL, nerve fiber layer; R, rod; C, cone; H, horizontal cells; B, bipolar cells; A, amacrine cells; G, RGCs; DSRGCs, direction selective retinal ganglion cells; J-RGCs, junctional adhesion molecule B-positive retinal ganglion cells; αRGCs, alpha RGCs; LED, local edge detector; iPRGCs, intrinsically photosensitive RGCs.

**Figure 4:**
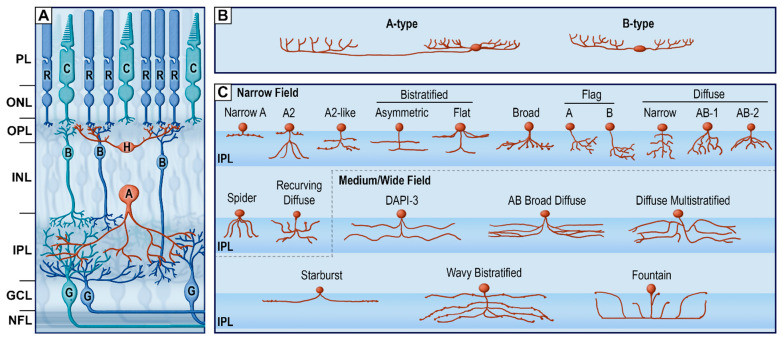
The retina horizontal pathway. (**A**) schematic organization of retinal cells. (**B**) Subtypes of horizontal cells. Redrawn from [60]. (**C**) Subtypes of narrow-field, medium-field and wide-field amacrine cells. Redrawn from [60,61]. PL, photoreceptor layer; ONL, outer nuclear layer; OPL, outer plexiform layer; INL, inner nuclear layer; IPL, inner plexiform layer; GCL, ganglion cell layer; NFL, nerve fiber layer; R, rod; C, cone; H, horizontal cells; B, bipolar cells; A, amacrine cells; G, retinal ganglion cells.

**Table 1:** The most common retinal diseases, from hereditary disorders to age- or trauma-related conditions, linked to their functional repercussions.

Pathology	Glare	VF Loss	Scotoma	Night Blindness	Light Adaptation	Nystagmus	Fluctuating Vision	Depth Perception
Achromatopsia	x				x	x		
Albinism	x				x	x		
Coloboma	x	x						
Diabetes	x	x	x		x		x	x
Glaucoma	x	x		x	x		x	x
Macular degeneration	x		x				x	x
Optic atrophy	x	x	x			x	x	x
Retinal detachment	x	x					x	
Retinopathy of prematurity	x	x	x				x	x
Retinitis pigmentosa	x	x		x	x		x	x
